# Meta-analysis of pain and function placebo responses in pharmacological osteoarthritis trials

**DOI:** 10.1186/s13075-019-1951-6

**Published:** 2019-07-15

**Authors:** ZeYu Huang, Jing Chen, Qin Sheng Hu, Qiang Huang, Jun Ma, Fu Xing Pei, Bin Shen, Virginia Byers Kraus

**Affiliations:** 1 Department of Orthopedic Surgery, West China Hospital, West China Medical School, Sichuan University, 37# Wainan Guoxue Road, Chengdu, Sichuan Province People’s Republic of China; 2 West China School of Stomatology, Sichuan University, Chengdu, Sichuan Province People’s Republic of China; 3 Division of Rheumatology, Department of Medicine, Duke University School of Medicine, Duke Molecular Physiology Institute, PO Box 104775, Room 51-205, Carmichael Building, 300 N Duke St., Durham, NC 27701-2047 USA

**Keywords:** Placebo response, Osteoarthritis, Treatment, Trial, Pain, Function, Outcome measures

## Abstract

**Objective:**

To evaluate contextual effects in the form of placebo responses (PRs) for patient-reported pain and function and objectively measured function in osteoarthritis (OA) clinical trials.

**Methods:**

Two authors independently searched major electronic databases from inception to 20 May 2019. Included studies were randomized, placebo-controlled OA trials of pharmacological agents reporting both patient-reported and objectively measured outcomes. PRs for each type of outcome measure were compared by standardized mean differences (SMDs). The placebo response ratio (PRR) assessed the placebo to treatment effect size. The effect sizes of PRs and PRRs were pooled using a random effects model.

**Results:**

Twenty-one trials met the inclusion criteria; 20 were double-blinded with one not reporting on blinding status. Compared with patients’ self-reported outcome (PRO) pain, PRs were significantly lower for PRO function (SMD − 0.16 [95% CI = − 0.28, − 0.05], *p* = 0.006), objectively measured muscle strength (SMD − 0.34 [95% CI − 0.58, − 0.10], *p* = 0.006), and range of motion (SMD = − 0.31 [95% CI = − 0.54, − 0.08], *p* = 0.008) function. Generally, PRs for function outcomes (patient-reported and objectively measured) were similar. The overall PRR for different measures ranged from the smallest (most favorable) for walking time/distance (0.30, 95% CI 0.16 to 0.43) to the largest for PRO pain (0.44, 95% CI 0.23 to 0.65).

**Conclusion:**

Function measures both subjective and objective had less contextual effects than pain measures in OA trials. Our results support the OMERACT-OARSI recommendations to include measures of physical function in all clinical trials of hip and knee OA and suggest that a greater use of function measures might enhance the success rates of pharmacological OA trials. Increasing the availability of mobile health apps should facilitate the acquisition of measured function data.

**Electronic supplementary material:**

The online version of this article (10.1186/s13075-019-1951-6) contains supplementary material, which is available to authorized users.

## Introduction

OA, the most common form of joint disease, affects ≥ 320 million individuals globally on the basis of age-standardized prevalence rate estimates. Aging promotes the development of OA in conjunction with other risk factors [1]. OA is a major cause of pain and disability; the risk of mobility disability (defined as needing help walking or climbing stairs) attributable to knee OA alone is greater than that attributable to any other medical condition in people aged 65 years and older [2]. Current treatments mainly focus on relieving pain and stiffness or improving function and quality of life. According to the guidance of the US Food and Drug Administration (FDA), an effective drug for OA is usually assessed by patients’ self-reported outcomes (PROs). However, high placebo responses (PRs) in OA trials are believed to have contributed to the long list of failures of OA trials to date. Based on emerging evidence from other placebo-controlled studies in other research fields [3, 4], we hypothesized that placebo responses in OA trials would be smaller (more favorable) for objective than for subjective measures.

PRs, once misunderstood as the effects of an “inert substance,” have been reported in the treatment of a wide range of conditions including pain [3], depression [5], asthma [6], hypertension [7], and irritable bowel syndrome [7], to name a few. Clinically, PRs are defined as improvements in patients’ symptoms that are attributable to their participation in the therapeutic process. These responses are distinct from those of discrete therapies and are perceived improvements in symptoms or overall health from the psychological effect of receiving treatment. Placebos can provide relief, but they rarely cure. Although many studies have demonstrated the objective pathways and the correlates of PRs, there is still a lack of evidence to show that the therapeutic benefits associated with PRs alter the pathophysiology of diseases beyond their symptomatic manifestations, ascertained as subjective and self-appraised symptoms. For instance, in the research field of cancer, there is no evidence that placebos can shrink tumors. However, common symptoms of cancer and side effects, such as fatigue, nausea, hot flashes, and pain, can be relieved by placebo treatments [8]. Wechsler et al. [9] have also shown that placebo treatment can dramatically relieve visual analog scale (VAS)-assessed pain but not improve patients’ forced expiratory volume in 1 s (FEV_1_). These results provided the impetus for this study, whose goal was to determine if there exists any difference in the strength of PRs for objective compared to subjective outcome measures in osteoarthritis (OA) clinical trials. Given the acceptance of measured function as a primary outcome for regulatory approval of drugs in other fields [10], we were particularly interested to determine the overall performance of objective function (with regard to PRs) relative to PRO function and PRO pain. Although utilized in the OA field, objective measures of function, such as six-min walk test, muscle strength and range of motion (ROM) have not, to our knowledge, been utilized as primary or co-primary outcomes in pharmacological OA trials. Specifically, our primary goal in this meta-analysis was to compare PRs for self-reported outcomes of pain and function to measured outcomes of pain and function in randomized, placebo-controlled pharmacological trials for OA of the knee, hip, foot, or hand. Secondarily, we compared PRs of pain measures to PRs of function measures.

## Methods

A systematic review (SR) and meta-analysis of placebo-controlled randomized clinical trials (RCTs) were performed using the approach recommended by Preferred Reporting Item for Systematic Review and Meta-Analyses (PRISMA) guidelines for meta-analysis of interventional studies. The review protocol was prospectively registered on PROSPERO (CDR42016049792).

### Search strategy and study selection

The following bibliographic databases were searched from inception to 20 May 2019: Medicine via PubMed, EMBASE via OVID, Web of Science, and the Cochrane Central Register of Controlled Trials. We searched free text and index terms related to “osteoarthritis”; “randomized, placebo-controlled trial”; and a specific treatment (e.g., paracetamol or acetaminophen) (see online supplementary search strategy). The reference list of the full-text articles, published SRs, and meta-analyses was also reviewed for additional eligible studies. No language limitation was applied. To be included in this analysis, studies had to meet the following criteria: (1) be randomized placebo-controlled trials; (2) include participants with OA of the knee, hip, foot, or hand; (3) compare placebo with active treatments including chondroitin, glucosamine, paracetamol or acetaminophen, oral non-steroidal anti-inflammatory drugs (NSAIDs), topical NSAIDs, intra-articular hyaluronic acid (IAHA), and/or intra-articular corticosteroid (IACS); (4) report patient-reported and objectively measured outcomes (i.e., PRO pain and/or function; measured pain and/or function); and (5) report changes from baseline and SDs or data from which these metrics could be derived.

### Quality assessment and data extraction

Two independent reviewers (QSH and JM) assessed the study quality or risk of bias in each study using the modified Jadad tool [11] in which allocation concealment was also assessed. Discrepancies between the two independent reviewers were resolved by consensus after a discussion, and a third reviewer was consulted if necessary (QH). Data were fully extracted and assessed by two investigators (ZYH and JC) and validated by a third investigator (QSH). Discrepancies were discussed and ratified by a senior investigator (BS). The extracted data included intervention description, inclusion/exclusion criteria, baseline data, values for all outcomes at baseline, post-intervention, and later follow-up. Items recorded were study design and setting, characteristics of participants (percentage of women, mean age), interventions (session, duration), and outcomes (at different time points). Repeated measurements of change from baseline and its SD were collected; if not presented, these were calculated from the outcomes at baseline and endpoint using a formula recommended by the Cochrane Collaboration that adjusts SD of the change score for the correlation between baseline and endpoint values [12]. The correlation coefficient was obtained from trials that reported SD of both baseline and endpoint measures and change from baseline. When more than one scale for the same PRO was reported (11 of 21 studies), for example, Western Ontario and McMaster Universities Arthritis Index (WOMAC) pain and VAS pain, the one with the lowest PR (effect size (ES) for the placebo) was selected to bias results in favor of PROs for the purposes of these analyses.

### Statistical analysis

The ES for each subgroup was calculated as mean change from baseline in units of its SD known as the standardized mean difference (SMD) [13]. The overall treatment response from baseline was defined as the ES of the active treatment group; the overall PR from baseline was defined as the ES of the placebo group. The PR ratio (PRR), defined as the ratio of total treatment response attributable to PR and its 95% CI, was calculated using the ES ratio between the PR and the overall treatment response [14]. Theoretically, the PRR should range from 0 (indicating no contribution from PR) to 1 (indicating 100% contribution from PR). When the ES of the PR was greater than that of the overall treatment response, the maximum value of 1 (100%) was assigned. Trials in which either the mean treatment or placebo group response worsened from baseline were excluded from the meta-analysis of PRR (*n* = 2 for PRO pain, *n* = 3 for PRO function, *n* = 0 for muscle strength, *n* = 8 for ROM) since (1) it may represent a side effect or nocebo response (negative placebo effect) that is not the focus of a PRR analysis and (2) the measure of PRR does not allow negative values, especially when the ratio is log transformed.

Meta-analyses were performed to determine the ES of PRs and overall treatment responses from the available pooled data using a random effects model. The PRRs were also pooled. The time point when the ES of the placebo group reached its lowest point was chosen for meta-analysis. The heterogeneity of studies was assessed using the *I*^2^ index test. Publication bias was accessed with funnel plots and a combination of Begg’s and Egger’s tests for analysis including more than 10 studies. Sensitivity analysis was performed to assess the effects of intervention blinding status; when blinding was not mentioned, the trial was considered an unblinded study (one study). A meta-regression was performed to evaluate the factors contributing to heterogeneity including the type of treatment (intra-articular vs. oral), year of the study, treatment duration, joint site, and sample size. All statistical analyses were performed using STATA Version 14 for Mac (StataCorp LP, TX, USA).

## Results

### Study selection and characteristics

**Fig. 1:**
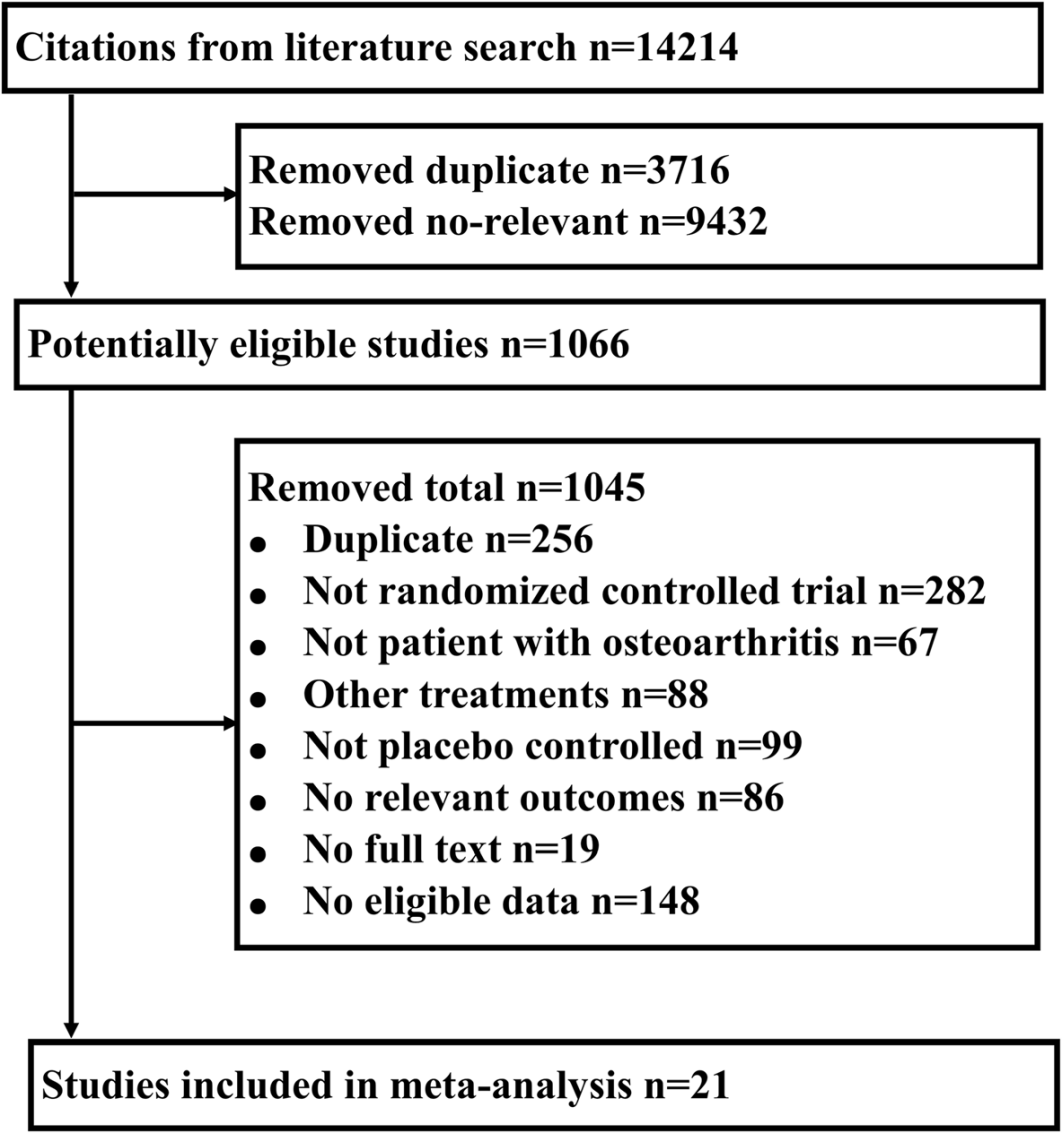
Flow diagram showing the screening process and search results

Figure 1 illustrates the process of selecting studies for this meta-analysis. In total, 14,214 potential studies were identified. Based on the title and abstract content, 13,148 of these studies were excluded. The full texts of the remaining 1066 studies were read, and a further 1045 were excluded, resulting in the retention of 21 studies in the qualitative and quantitative synthesis of this review. Of note, no studies were identified that included measured pain outcomes simultaneous with PRO pain and/or function outcomes. A total of 2162 patients were included: 1066 patients in placebo groups and 1096 patients in the treatment groups. The characteristics of the included studies are listed in Additional file 5: Table S1. The methodological quality of all 21 included studies was high (> 4 points) (Additional file 6: Table S2) [15–35]. All outcomes with data appropriate for this report were extracted and included in the meta-analysis. Outcome measures were grouped according to their construct and design (Additional file 7: Table S3 and Additional file 8: Table S4).

### Meta-analysis of PR

#### PRO pain vs. PRO function

**Fig. 2:**
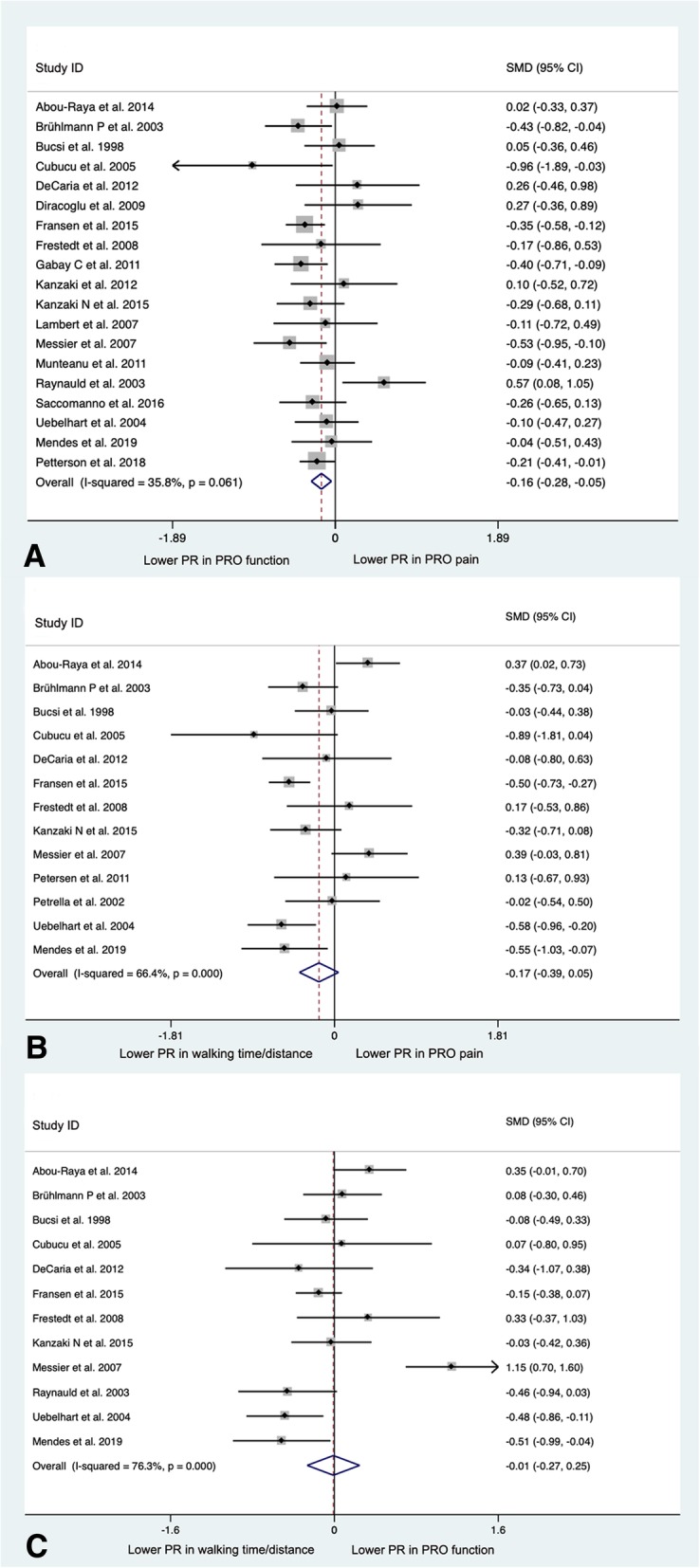
**a** Forest plot analysis of the comparison between PRO pain and PRO function. **b** Forest plot analysis of the comparison between PRO pain and walking time/distance. **c** Forest plot analysis of the comparison between PRO function and walking time/distance. (PRO, patient-reported outcomes; PR, placebo response; SMD, standardized mean difference; CI, confidence interval)

Among the 21 included studies, 19 trials [15–18, 20–25, 27–35] provided data on both PRO pain and PRO function. Based on SMD, the PR of PRO function was significantly lower than that of PRO pain (SMD = − 0.16 [95% CI = − 0.28, − 0.05], *p* = 0.006, *I*^2^ = 35.8%) (Fig. 2a).

#### PRO pain vs. measured function

All 21 included studies provided data on both PRO pain and measured function (measured muscle strength, walking time/distance, or ROM). Based on the four studies [19, 20, 29, 32] providing data on both PRO pain and muscle strength, the PR was significantly lower for PRO muscle strength than PRO pain (SMD = − 0.34 [95% CI = − 0.59, − 0.10], *p* = 0.006, *I*^2^ = 33.8%) (Additional file 1: Figure S1A). Based on the 13 studies providing data on both PRO pain and walking time/distance [15–21, 25–27, 31, 33, 34], the PR for walking time/distance was lower but the difference was not statistically significant (SMD = − 0.17 [95% CI = − 0.39, 0.05], *p* = 0.121, *I*^2^ = 66.4%) (Fig. 2b). Based on the 8 studies [22–24, 28–30] providing data on both PRO pain and ROM (measured by clinical examination performed by assessors blinded to the patient treatment assignment), the PR was significantly lower for measured ROM than for PRO pain (SMD = − 0.31 [95% CI = − 0.54, − 0.08], *p* = 0.008, *I*^2^ = 57.3%) (Additional file 1: Figure S1B).

#### PRO function vs. measured function

Among the 21 included studies, 19 studies provided data on both PRO function and measured function (measured ROM, walking time/distance, or muscle strength). Based on the 8 studies [19, 22–24, 28–30, 34] providing data on both PRO function and ROM, the PR was significantly lower for measured ROM than for PRO function (SMD = − 0.43 [95% CI = − 0.70, − 0.15], *p* = 0.002, *I*^2^ = 69.8%) (Additional file 1: Figure S1C). Based on the 12 studies providing data on both PRO function and walking time/distance [15–18, 20, 21, 23, 25, 27, 31, 33, 34], there was no significant difference of the PR for walking time/distance and PRO function (SMD = − 0.01 [95% CI = − 0.27, 0.25], *p* = 0.945, *I*^2^ = 76.3%) (Fig. 2c). Based on the 3 studies [20, 29, 36] providing data on both PRO function and muscle strength, there was no significant difference of the PR for muscle strength and PRO function (SMD = − 0.06 [95% CI = − 0.25, 0.13], *p* = 0.55, *I*^2^ = 0%) (Additional file 1: Figure S1D).

### Meta-analysis of PRR

**Fig. 3:**
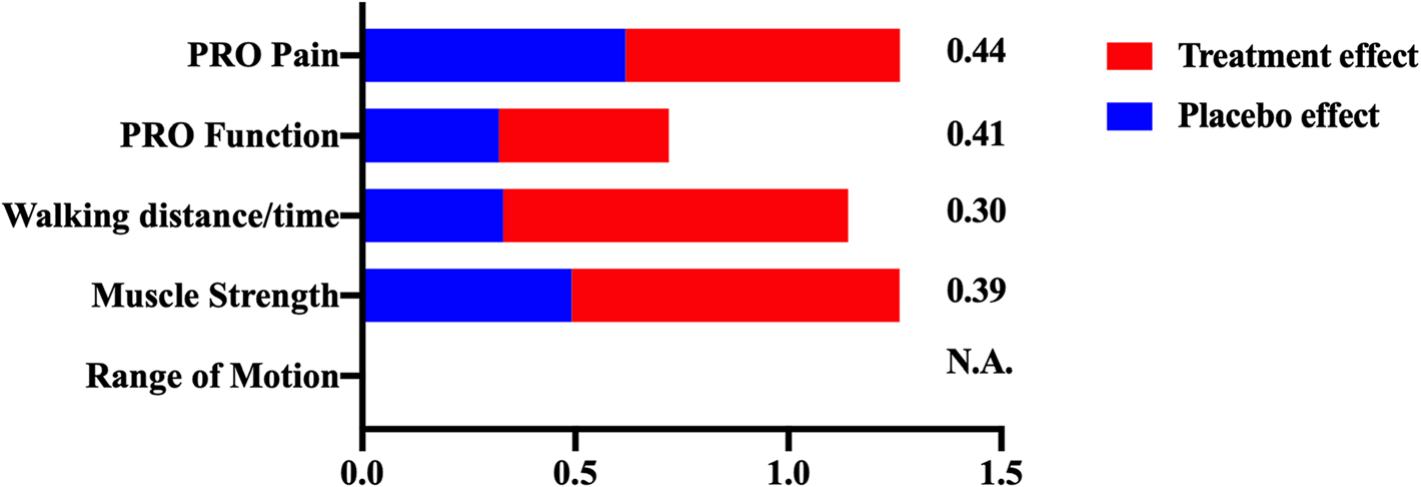
The overall treatment response and the placebo response ratio for different measures in osteoarthritis. From the lowest (most favorable placebo response relative to treatment effect) to the highest (least favorable placebo response relative to treatment effect) were 0.30 (95% CI 0.16 to 0.43) for walking time/distance, 0.39 (95% CI 0.09 to 0.68) for muscle strength, 0.41 (95% CI 0.26 to 0.57) for PRO function, and 0.44 (95% 0.23 to 0.65) for PRO pain. (PRO, patient-reported outcomes; PRR, placebo response ratio; N.A, none available)

To determine whether the differences in placebo response rates of pain and function, subjective and objective outcomes, translate into differences in effect sizes of treatment responses, we evaluated the placebo response ratio (PRR, the PR relative to treatment response). The PRRs, from the lowest (most favorable placebo response relative to treatment effect) to the highest (least favorable placebo response relative to treatment effect), were 0.30 (95% CI 0.16 to 0.43) for walking time/distance, 0.39 (95% CI 0.09 to 0.68) for muscle strength, 0.41 (95% CI 0.26 to 0.57) for PRO function, and 0.44 (95% CI 0.23 to 0.65) for PRO pain (Fig. 3). A meta-analysis of PRRs for ROM was not possible because either the treatment or placebo groups worsened from baseline for this outcome (Fig. 3).

### Publication bias analysis

Three comparisons included more than 10 studies; for these analyses, there were only slight asymmetrical distributions of published studies in the funnel plots (Additional file 2: Figure S2). Based on Egger’s and Begg’s tests, there was no evidence of publication bias for the comparisons of the following: PRO pain and walking time/distance (Egger’s *p* = 0.415, Begg’s *p* = 0.583), PRO function and walking time/distance (Egger’s *p* = 0.748, Begg’s *p* = 0.945), or PRO pain and PRO function (Egger’s *p* = 0.217, Begg’s *p* = 0.263).

### Sensitivity analysis related to participant blinding

Of the 21 studies, 20 were double-blinded; only one study did not report on the blinding status of the participants. Sensitivity analyses demonstrated no effect on the PR and PRR results with elimination of one study for which blinding was not reported (Additional file 3: Figure S3).

### Meta-regression to evaluate factors contributing to heterogeneity

Of the features evaluated (type of treatment, year of the study, treatment duration, joint site, and sample size), only the type of treatment contributed to the heterogeneity of results explaining 69.34% of the heterogeneity (*p* = 0.011). In general, both PRO function and PRO pain responses were higher in intra-articular studies than in oral pharmacological studies. A subgroup analysis of the type of treatment revealed a statistically significantly lower PR for PRO function compared to PRO pain in oral pharmacological studies but not in intra-articular treatments (Additional file 4: Figure S4).

## Discussion

Although there is a large amount of literature on PR, the PRs for different measures are rarely compared. This study focused on the comparison of PR between pain and function, and measured and self-reported outcomes in OA trials. Many different OA treatments have been formally tested in randomized placebo-controlled trials, allowing an excellent opportunity to explore the PRs for different measures. The current meta-analysis yielded two key findings: (a) For minimizing PRs using self-reported measures alone, PRO function showed a significant advantage over PRO pain; (b) Compared with self-reported measures, objective measures had equal or lower PRs. For instance, all objective measures (muscle strength, ROM, and walking time/distance) had lower PRs than PRO pain; these results were significant for muscle strength and ROM. Similarly, comparing PRO function to measured function, the measured outcomes tended to have equal or lower PRs than PRO function (ROM was superior while walking distance/time and muscle strength were comparable). Our observation that PRO symptoms yielded the highest PRs is consistent with previous studies in OA and other conditions. Previously, Zhang et al. [37] reported that objective outcomes, such as quadriceps strength, joint space width, and ROM, tended to have lower PRs than PRO pain. These data show there are greater contextual effects for pain than for function.

Whether PRs only occur for subjective measures remains controversial [6, 36]. A landmark meta-analysis that evaluated PRs across multiple conditions showed significantly higher PRs of subjective (PROs) than objective measures [3, 38]. Strikingly consistent observations have emerged from asthma studies demonstrating significantly higher PRs for patient-reported subjective measures, such as symptom severity and asthma control scores, compared to objective measures, such as FEV_1_, peak flow, and maximal mid-expiratory flow [9, 39]. Meta-analyses of trials of anti-hypertensives and anti-hyperglycemics showed a small but steady increase in PRs over the last 18–20 years despite the use of the objective outcomes including change in measured blood pressure [40] and change in HbA1c [41], respectively; interestingly, these studies showed that reductions in blood pressure and HbA1c with drug treatments increased in parallel by the same amounts as changes with placebo resulting in consistent drug effect sizes over time. These results from many disease areas demonstrate that PRs exist for both subjective and objective measures, although PRs for objective are consistently lower than subjective measures. Nevertheless, some researchers argued that cross-disease comparisons are problematic; therefore, studies should be conducted on different outcome measures in specific conditions [37].

Many previous studies have shown that OA trials tend to have high PRs [37, 42, 43]. This phenomenon poses a challenge for clinical trial design and is believed to have blocked the successful development of therapeutics for OA [43]. All these factors provide a strong rationale for this study. The challenge posed by PRs in clinical trials is not the magnitude of the PR but rather the magnitude of the PR relative to the treatment effect. A previous meta-analysis of OA trials showed a rise in the PR of the placebo group in proportion to the ES of the active treatment, an effect known as the “placebo analgesia” theory [44]. In our study, the lower PRs for objective measures compared to subjective measures could arise in the context of correspondingly lower treatment effects for objective measures relative to subjective measures. For this reason, we evaluated, when possible, the PRRs in the included studies. The concept of PRR is similar to the proportion attributable to contextual effects (PCE) [45], to determine how much of an overall treatment effect could be attributed to a PR. Consistent with our PR results, the PRRs were higher for subjective compared to objective measures suggesting higher placebo responses relative to treatment effects for subjective compared with objective measures in OA trials.

Compared to published PCEs [45], we observed lower PRs relative to treatment effects in our study. There might be several reasons for this difference. Firstly, we only included studies of pharmacological agents (oral, topical, intra-articular, or patch), while Zou et al. [45] included more invasive treatments, such as lavage, with a PCE of 0.91. In a systematic review of 53 studies, Wartolowska et al. [46] reported higher PRs for more invasive procedures, such as surgery with a mean PR rate of 74%. Consistent with Zou et al. [45], Doherty and Dieppe [47] reported a stronger PR for invasive than non-invasive procedures. Secondly, the current study selected the PRO with the lowest placebo response when more than one scale for the type of measure (i.e., pain) was available; this was intended to bias the comparison in favor of subjective measures presented “at their best” vs. objective measures but could result in lower PRR than PCE, for which such selection was not done.

Our data search revealed that few OA studies have ever reported objectively measured pain outcomes [48, 49] and none reported PRO pain relative to objectively measured pain. This might be partly attributed to the lack of an established threshold for objectively measured pain and only moderate correlations of objectively measured pain and PRO pain [49]. To address this knowledge gap, more studies are needed to validate objective measures of pain and determine their clinical relevance in OA.

Interestingly, PRO function had a significantly lower PR than PRO pain in the current study. To our knowledge, this finding has never been reported previously in the field. We believe there might be two explanations for this finding. Firstly, compared with self-reported pain, self-reported function tends to correlate with performance-based function and muscle strength. For instance, Park et al. [50] found that the WOMAC functional scale had a more significant correlation with actual joint muscle strength than pain scores. Zeni et al. [51] also reported that PRO function (Hip Outcome Score in end-stage hip OA) had a strong correlation with both performance-based function and muscle strength, in contrast to PRO pain that had no correlation with muscle strength. This is also consistent with our finding for comparable results for PRO function and measured (walking time/distance and muscle strength) function. Secondly, chronic joint pain can be nociceptive, neuropathic, and augmented by central sensitization [52]. Comorbidities such as anxiety and depression may exacerbate pain sensations [53]. According to Bryant [54], for patients experiencing chronic pain, psychological conditions interact with autobiographical memory to cause an overestimation of pain as assessed by self-reported PROs. Taken together, the complex etiologies of pain are reflected in pain PROs, and not all aspects would be expected to be treated uniformly by a drug.

Some limitations of the current meta-analysis warrant discussion. First and foremost, the PR was largely determined as the difference between baseline and endpoint, rather than the difference in benefit between the placebo and non-treatment (observation only) groups. Secondly, although there are many treatments for OA, such as pharmacological, non-pharmacological, surgical, and complementary treatments, based on the limited number of studies reporting on objective function measures, we were only able to examine a limited selection of pharmacological interventions rather than all treatments for OA. Some of the included treatments are largely considered minimally effective to ineffective for the treatment of OA. Thirdly, due to the lack of reporting in one study, we could not be sure that all participants in the analysis were blinded to treatment; this might cause differences between unblinded patients and blinded ones in their way of reporting treatment responses. Some important treatments for OA, such as exercise, patient education, and change of lifestyle, are not readily amenable to a blinded placebo or sham intervention. In the absence of a blinded placebo-controlled randomized study, it is very difficult to estimate the true PR for different measures. For this reason, we focused on pharmacological trials for this meta-analysis, the majority (20 of 21) of which blinded the trial participants; sensitivity analysis demonstrated no difference in the results when the one possibly unblinded study was eliminated. Fourthly, we only included 21 RCTs in this meta-analysis because of the requirement for the specified outcome measures. Compared to the large volume of clinical trials in OA, the dataset was relatively limited. Fifthly, the pooled results of four PRRs showed big overlaps among the 95% CIs. Although the characteristics of the data do not allow testing for statistically significant differences among the four PRRs, we speculate that incremental improvements in the trial design, in the form of lower PRs and/or lower PRRs, may increase the likelihood of observing a treatment effect and/or decrease the sample size needed to show a treatment effect. The routine adoption of objective outcome measures might provide stable effect sizes over time, despite varying PRs as demonstrated by recent meta-analyses of trials of anti-hypertension and anti-hyperglycemic drugs [40, 41].

It is important to emphasize that high heterogeneity was detected in several comparisons due to the variance among trials in methods, imputation of missing data, disease stage, study duration, and/or random variation [55]. To overcome this, a random effects model was chosen. In addition, it is widely recognized that negative trials are less likely to be published. Our analyses were limited to published and publicly available RCTs; therefore, estimates of PRs based on the published literature may be less than if all RCTs could be examined [56].

## Conclusions

Our findings from published OA trials indicate that objective measures, such as walking time/distance, muscle strength, and ROM, have an equal or lower PR compared to subjective measures, such as PRO pain and PRO function. Moreover, for subjective measures alone, PRO function measures had lower PRs than PRO pain. In summary, function measures, both subjective and objective, were subject to less contextual effects in the form of placebo responses than pain measures in OA trials. Our results support the latest OMERACT-OARSI recommendation of a set of core domains that include measures of physical function in all clinical trials of hip and knee OA [57], and suggest that a greater use of function measures might enhance the success rates of pharmacological OA trials. The increasing availability and popularity of mobile health apps [58] should facilitate the acquisition of measured function data.

## Additional files

Additional file 1: **Figure S1.** A. Forest plot analysis of the comparison between PRO pain and muscle strength. B. Forest plot analysis of the comparison between PRO pain and ROM. C. Forest plot analysis of the comparison between PRO function and ROM. D. Forest plot analysis of the comparison between PRO function and muscle strength. (PRO = patient-reported outcomes; PR = placebo response; ROM = range of motion; SMD = standardized mean difference; CI = confidence interval). (TIF 2908 kb)

Additional file 2: **Figure S2.** A. Funnel plot of the comparison between PRO pain and function. B. Funnel plot of the comparison between PRO pain and walking time/distance. C. Funnel plot of the comparison between PRO function and walking time/distance. (PRO = patient-reported outcomes; SMD =standardized mean difference; s.e. = standard error; SMD = standardized mean difference). (TIF 1551 kb)

Additional file 3: **Figure S3.** A. Sensitivity analysis of the comparison between PRO pain and function (the one study that did not report on blinding status was excluded). B. Sensitivity analysis of the comparison between PRO pain and walking time/distance (the one study that did not report on blinding status was excluded). C. Sensitivity analysis of the comparison between PRO function and walking time/distance (the one study that did not report on blinding status was excluded). (PRO = patient-reported outcomes; PR = placebo response; SMD = standardized mean difference; CI = confidence interval). (TIF 3061 kb)

Additional file 4: **Figure S4.** A. Forest plot analysis of the comparison between PRO pain and PRO function (subgroup analysis based on whether studies used invasive treatment or not). (PRO = patient-reported outcomes; PR = placebo response; SMD = standardized mean difference; CI = confidence interval). (TIF 767 kb)

Additional file 5: **Table S1.** General information on studies in the meta-analysis. (DOCX 20 kb)

Additional file 6: **Table S2.** Summary of methodological quality based on the modified Jadad tool. (DOCX 17 kb)

Additional file 7: **Table S3.** Summary of the outcomes reported in the included studies. (DOCX 15 kb)

Additional file 8: **Table S4.** Summary of standardized instruments tools used for patient-reported pain and function in the included studies. (DOCX 19 kb)

## Data Availability

Please contact the authors for data requests.

## Abbreviations

CI: Confidence interval
ES: Effect size
FDA: Food and Drug Administration
FEV_1_: Forced expiratory volume in 1 s
IACS: Intra-articular corticosteroid
IAHA: Intra-articular hyaluronic acid
NSAIDs: Non-steroidal anti-inflammatory drugs
OA: Osteoarthritis
PCE: Proportion attributable to contextual effects
PRISMA: Preferred Reporting Item for Systematic Review and Meta-Analyses
PRO: Patients’ self-reported outcome
PRR: Placebo response ratio
PRs: Placebo responses
RCTs: Randomized clinical trials
ROM: Range of motion
SD: Standard deviation
SMDs: Standardized mean differences
SR: Systematic review
VAS: Visual analog scale
WOMAC: Western Ontario and McMaster Universities Arthritis Index
