# Supplementary figures and images for: Meta-analysis of pain and function placebo responses in pharmacological osteoarthritis trials
Source: Arthritis Res Ther. 2019 Jul 15;21:173. doi: 10.1186/s13075-019-1951-6 (PMC6631867; doi:10.1186/s13075-019-1951-6)

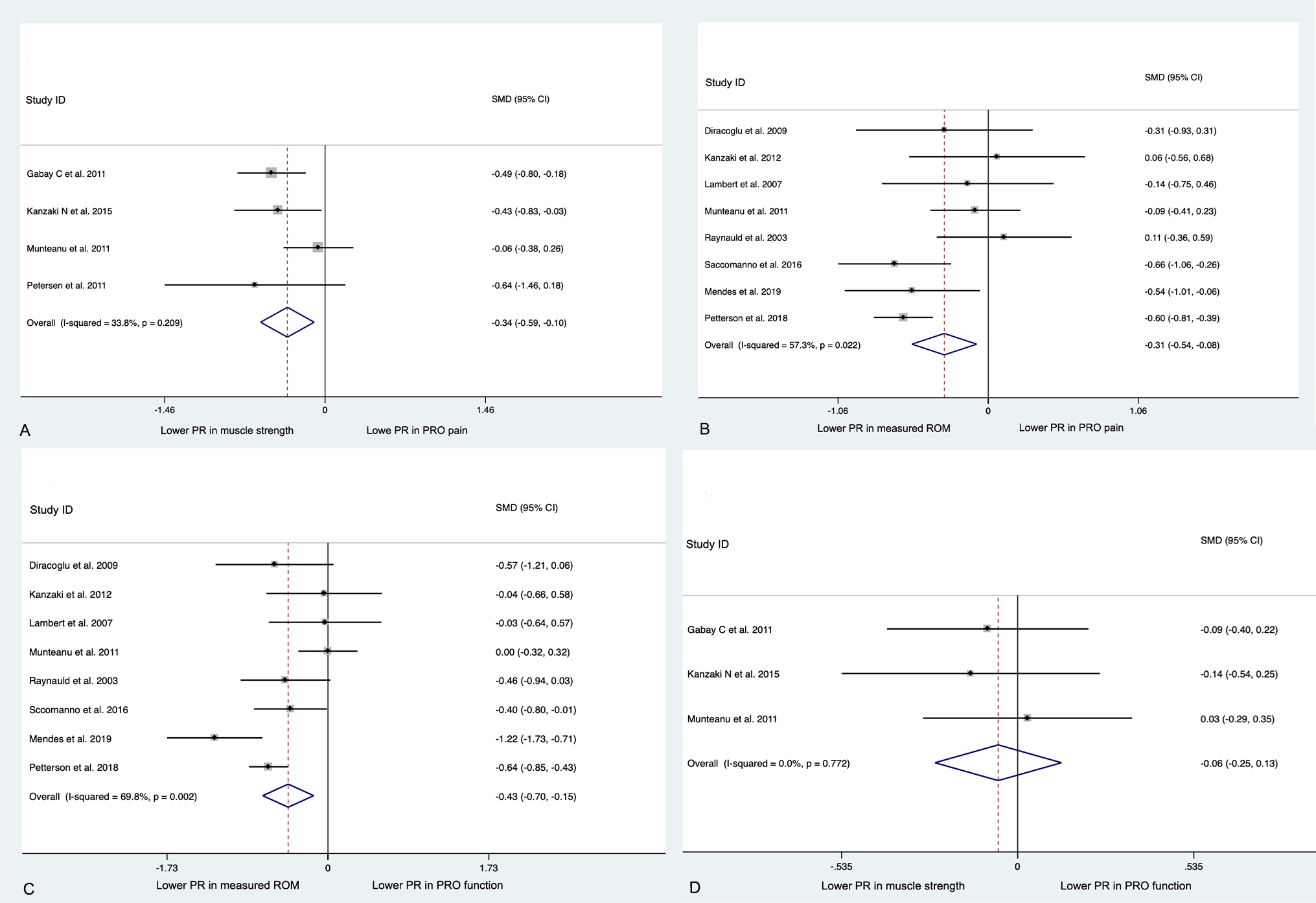

Supplement: Supplementary file 1 — Figure S1. A. Forest plot analysis of the comparison between PRO pain and muscle strength. B. Forest plot analysis of the comparison between PRO pain and ROM. C. Forest plot analysis of the comparison between PRO function and ROM. D. Forest plot analysis of the comparison between PRO function and muscle strength. (PRO = patient-reported outcomes; PR = placebo response; ROM = range of motion; SMD = standardized mean difference; CI = confidence interval). (TIF 2908 kb) [file 13075_2019_1951_MOESM1_ESM.tif]

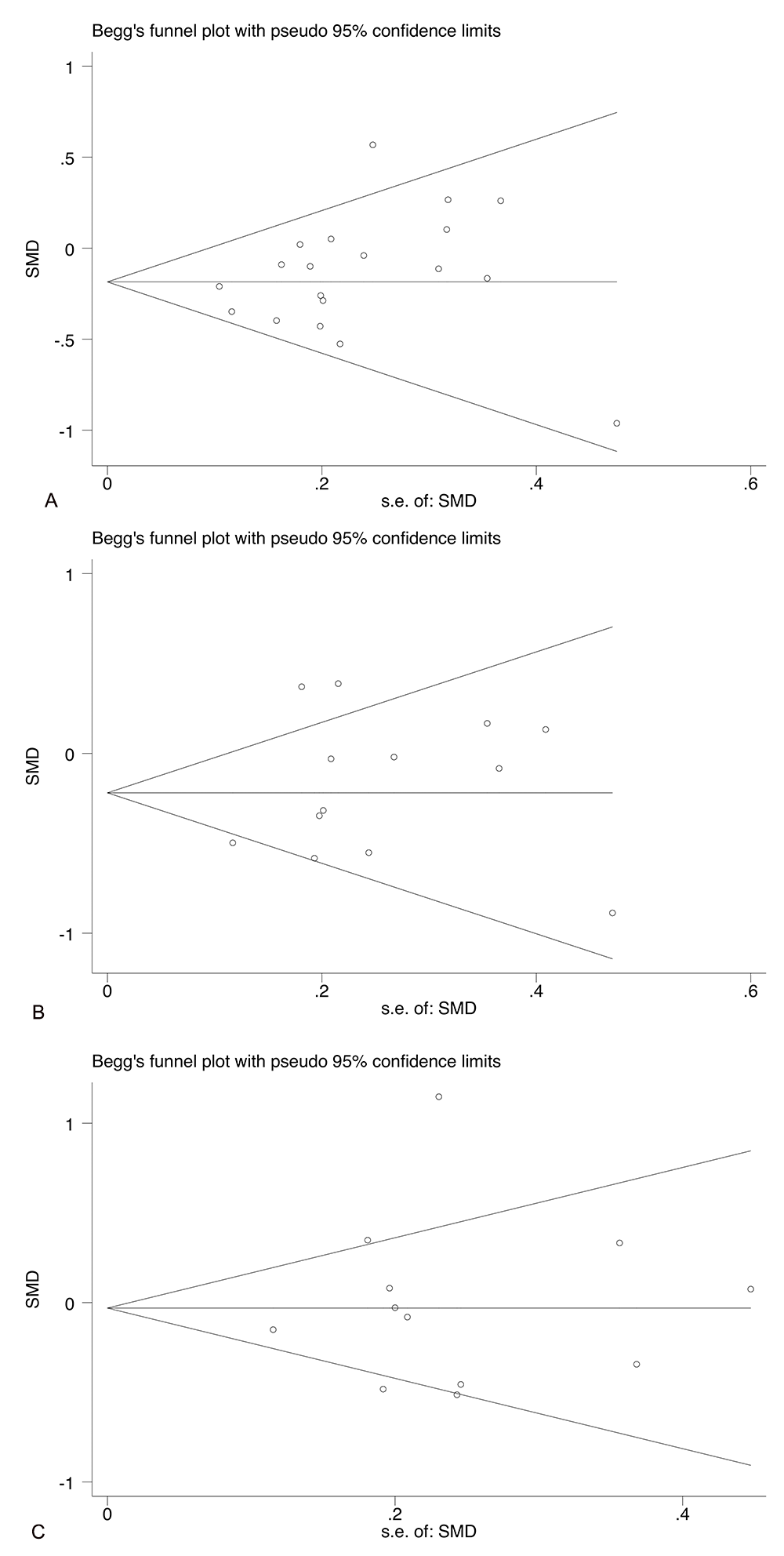

Supplement: Supplementary file 2 — Figure S2. A. Funnel plot of the comparison between PRO pain and function. B. Funnel plot of the comparison between PRO pain and walking time/distance. C. Funnel plot of the comparison between PRO function and walking time/distance. (PRO = patient-reported outcomes; SMD =standardized mean difference; s.e. = standard error; SMD = standardized mean difference). (TIF 1551 kb) [file 13075_2019_1951_MOESM2_ESM.tif]

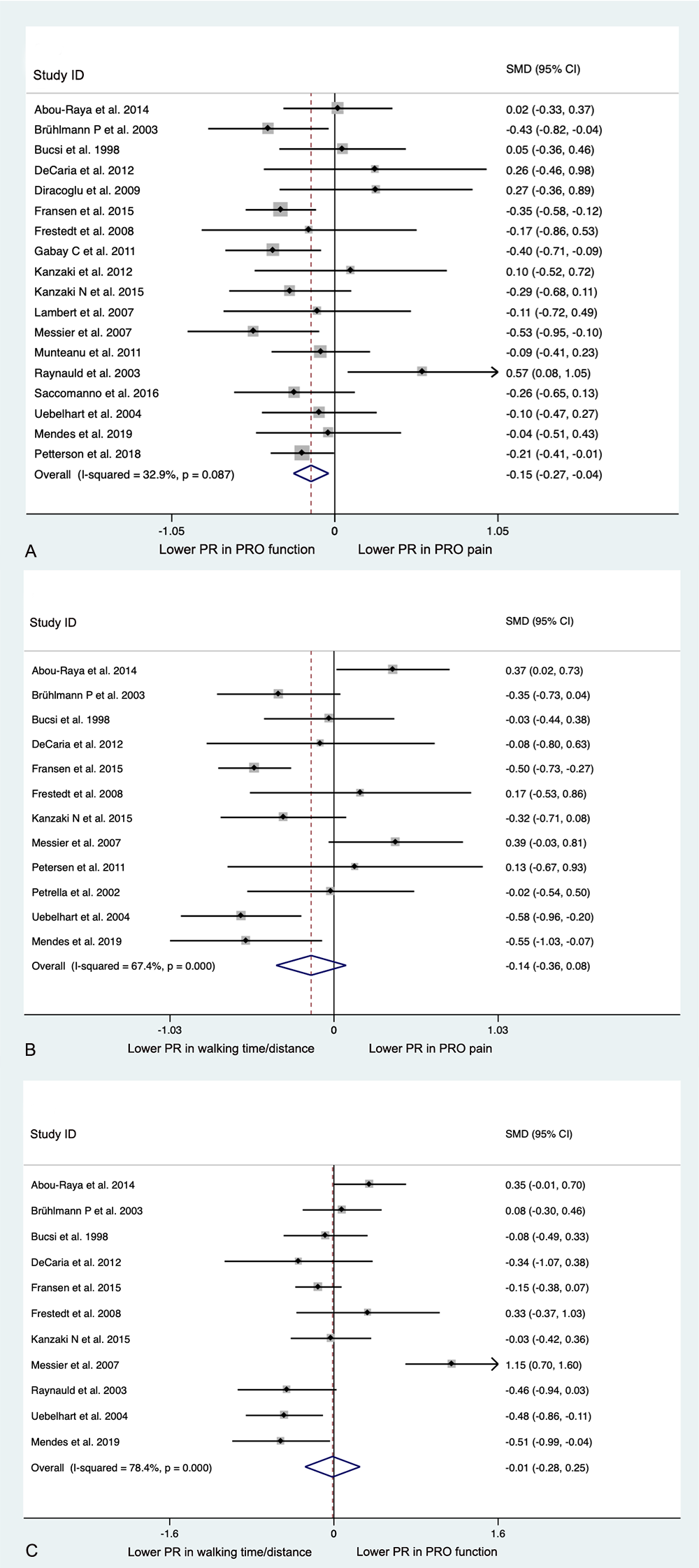

Supplement: Supplementary file 3 — Figure S3. A. Sensitivity analysis of the comparison between PRO pain and function (the one study that did not report on blinding status was excluded). B. Sensitivity analysis of the comparison between PRO pain and walking time/distance (the one study that did not report on blinding status was excluded). C. Sensitivity analysis of the comparison between PRO function and walking time/distance (the one study that did not report on blinding status was excluded). (PRO = patient-reported outcomes; PR = placebo response; SMD = standardized mean difference; CI = confidence interval). (TIF 3061 kb) [file 13075_2019_1951_MOESM3_ESM.tif]

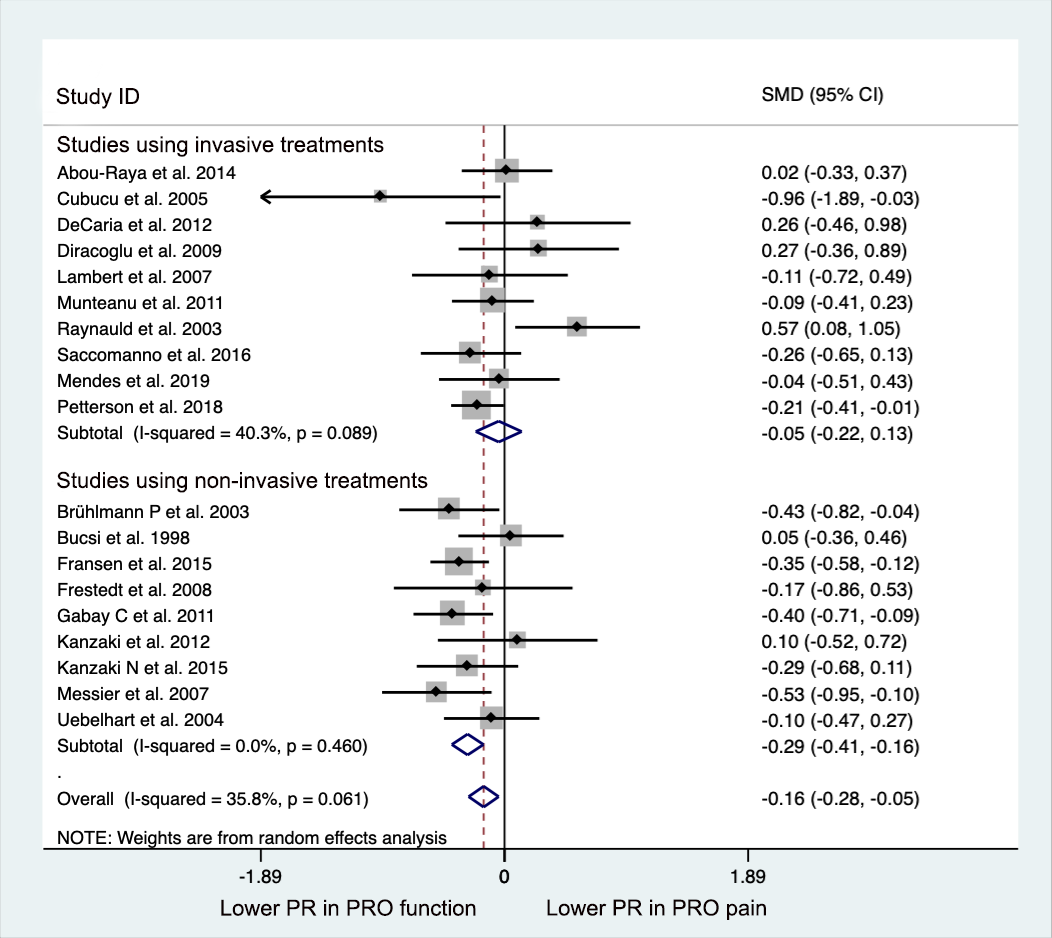

Supplement: Supplementary file 4 — Figure S4. A. Forest plot analysis of the comparison between PRO pain and PRO function (subgroup analysis based on whether studies used invasive treatment or not). (PRO = patient-reported outcomes; PR = placebo response; SMD = standardized mean difference; CI = confidence interval). (TIF 767 kb) [file 13075_2019_1951_MOESM4_ESM.tif]
