# Supplementary material for: Meta-analysis of pain and function placebo responses in pharmacological osteoarthritis trials
Source: Arthritis Res Ther. 2019 Jul 15;21:173. doi: 10.1186/s13075-019-1951-6 (PMC6631867; doi:10.1186/s13075-019-1951-6)
Supplement: Supplementary file 5 — Table S1. General information on studies in the meta-analysis. (DOCX 20 kb) [file 13075_2019_1951_MOESM5_ESM.docx]

**Supplemental Table 1. General information on studies in the meta-analysis.**

| Study | Diagnosis | Criteria for diagnosis | Intervention | Dose | Follow-up | ITT Analysis | No. of patient in placebo group | Baseline Characteristics | | | Withdraw rate |
| --- | --- | --- | --- | --- | --- | --- | --- | --- | --- | --- | --- |
|  |  |  |  |  |  |  |  | Mean age (y) | Female (%) | BMI (kg/m^2^) |  |
| Bucsi et al. 1998 | Knee OA | X-ray | Chondroitin Sulfate | 800mg/d po. | 6 months | Yes | 46 | 59.4±9.0 | 63% | 27.25 | 4.35% |
| Uebelhart  et al. 2004 | Knee OA | ACR criteria | Chondroitin Sulfate | 800mg/d po. | 12 months | Yes | 56 | 63.7±8.1 | 82.1% | / | 26.8% |
| Fransen  et al. 2015 | Knee OA | ACR criteria | Chondroitin Sulfate | 800mg/d po | 24 months | Yes | 151 | 60.6±8.1 | 54% | 29.1±5.8 | 22.52% |
| Frestedt  et al. 2008 | Knee OA | ACR criteria | Glucosamine Sulfate | 1500mg/d po. | 12 weeks | Yes | 16 | 58.9±7.4 | 62.5% | 32.4 | 43.75% |
| Petersen  et al. 2011 | Knee OA | ACR criteria | Glucosamine | 1500mg/d po. | 12 weeks | No | 12 | 63.1±4.7 | 58.3% | 28.3±3.2 | 0% |
| Kanzaki N  et al. 2015 | Knee OA | JOA criteria | Glucosamine  Chondroitin sulfate  Type II Collagen peptide  Quercetin glycosides Imidazole peptides  Vitamin D | 1200mg/d po.  60mg/d po.  45mg/d po.  90mg/d po.  10mg/d po.  5mg/d po. | 16 weeks | No | 50 | 51.6±1.1 | 56% | 23.3±0.5 | 18% |
| Messier  et al. 2007 | Knee OA | ACR criteria | Glucosamine Sulfate  Chondroitin Sulfate | 1500mg/d po.  1200mg/d po. | 12 months | Yes | 44 | 74.1±1.32 | 65.9% | 27.3±0.71 | 20% |
| Kanzaki  et al. 2012 | Knee OA | JOA criteria | Glucosamine Sulfate  Chondroitin Sulfate  Quercetin | 1200mg/d po.  60mg/d po.  45mg/d po. | 16 weeks | No | 20 | 58.3±7.4 | 85% | 22.0±3.4 | 0% |
| Raynauld  et al. 2003 | Knee OA | ACR criteria | IA steroid  Triamcinolone acetonide | 40mg | 24 months | Yes | 34 | 63.3±9.0 | 61% | / | 2.9% |
| Lambert  et al. 2007 | Hip OA | ACR criteria | IA steroid  Triamcinolone acetonide | 40mg | 6 months | Yes | 21 | 56.9±11 | 68% | / | 9.52% |
| Abou-Raya  et al. 2014 | Knee OA | ACR criteria | Oral prednisolone | 7.5mg/d | 6 weeks | Yes | 62 | 68.0±5.8 | 90.3% | 27.5±5.1 | 4.84% |
| Petrella  et al. 2002 | Knee OA | ACR criteria | IA HA | 2mL*10mg/mL  Weekly for 3 weeks | 4 weeks | Yes | 28 | 62.6±9.5 | 42.8% | 29.5±4.2 | 6.67% |
| Cubucu  et al. 2005 | Knee OA | ACR criteria | IA HA | 2mL*10mg/mL  Weekly for 3 weeks | 8 weeks | No | 10 | 57.6±2.77 | 100% | 26.78 | 0% |
| Diracoglu  et al. 2009 | Knee OA | ACR criteria | IA HA | 2mL*10mg/mL  Weekly for 3 weeks | 3 weeks | No | 20 | 56.2±7.2 | 100% | 31.3±4.0 | 4.76% |
| Munteanu  et al. 2011 | Foot OA | X-ray | IA HA | 1mL G-F 20  Monthly for 3 months | 6 months | Yes | 76 | 55.3±11.2 | 36.8% | 27.2±3.9 | 7.89% |
| Saccomanno et al. 2016 | Knee OA | ACR criteria | IA HA | 2mL*15mg/mL  One injection each 2 weeks  Three injections | 6 months | Yes | 51 | 61.2±10.1 | 64.7% | 27.5 (25-29.8) | 7.27% |
| DeCaria JE  et al. 2012 | Knee OA | ACR criteria | IA HA | 2mL* 20mg/mL  Weekly for 3 weeks | 6 months | Yes | 15 | 72.93±5.48 | 47% | 29.4±4.11 | 0% |
| Gabay C et al. 2011 | Hand OA | ACR criteria | Glucosamine Sulfate | 800mg/d po. | 6 months | Yes | 82 | 63±7.2 | 75.6% | 25.0±3.9 | 18.3% |
| Brühlmann P et al. 2003 | Knee OA | X-ray | DHEP-patch | Twice a day  14 days | 14 days | Yes | 52 | 64.8±10.6 | 64% | 28.9 | 3.8% |
| Mendes et al.  2019 | Knee OA | ACR criteria | IA steroid  Triamcinolone acetonide | 40mg | 12 weeks | Yes | 35 | 64.6±6.7 | 94.3% | 30.5±5.3 | 0% |
| Petterson et al.  2018 | Knee OA | ACR criteria | IA HA | 4mL Monovisc^TM^ | 26 weeks | Yes | 185 | 58.7 ± 9.2 | 57.3% | 30.4 ± 4.6 | 8.6% |

OA: Osteoarthritis; ITT: intention to treat; ACR: American College of Rheumatology; JOA: Japanese Orthopedic Association; BMI: Body Mass Index; IA: Intra-articular; HA: Hyaluronic acid;
