# Supplementary material for: Meta-analysis of pain and function placebo responses in pharmacological osteoarthritis trials
Source: Arthritis Res Ther. 2019 Jul 15;21:173. doi: 10.1186/s13075-019-1951-6 (PMC6631867; doi:10.1186/s13075-019-1951-6)
Supplement: Supplementary file 6 — Table S2. Summary of methodological quality based on the modified Jadad tool. (DOCX 17 kb) [file 13075_2019_1951_MOESM6_ESM.docx]

**Supplemental Table 2. Summary of methodological quality based on modified Jadad tool.**

| Study | Item | | | | | | | | Total |
| --- | --- | --- | --- | --- | --- | --- | --- | --- | --- |
|  | 1 | 2 | 3 | 4 | 5 | 6 | 7 | 8 |  |
| Bucsi et al. 1998 | 🗸 | 🗴 | 🗸 | 🗴 | 🗸 | 🗸 | 🗴 | 🗸 | 5 |
| Uebelhart et al. 2004 | 🗸 | 🗸 | 🗸 | 🗴 | 🗸 | 🗸 | 🗸 | 🗸 | 7 |
| Fransen et al. 2015 | 🗸 | 🗸 | 🗸 | 🗸 | 🗸 | 🗸 | 🗸 | 🗸 | 8 |
| Frestedt et al. 2008 | 🗸 | 🗴 | 🗸 | 🗴 | 🗸 | 🗸 | 🗸 | 🗸 | 6 |
| Petersen et al. 2011 | 🗸 | 🗸 | 🗸 | 🗴 | 🗸 | 🗸 | 🗴 | 🗸 | 6 |
| Kanzaki N et al. 2015 | 🗸 | 🗸 | 🗸 | 🗴 | 🗸 | 🗸 | 🗸 | 🗸 | 7 |
| Messier et al. 2007 | 🗸 | 🗴 | 🗸 | 🗴 | 🗸 | 🗸 | 🗸 | 🗸 | 6 |
| Kanzaki et al. 2012 | 🗸 | 🗸 | 🗸 | 🗴 | 🗴 | 🗸 | 🗸 | 🗸 | 6 |
| Raynauld et al. 2003 | 🗸 | 🗸 | 🗸 | 🗸 | 🗸 | 🗸 | 🗸 | 🗸 | 8 |
| Lambert et al. 2007 | 🗸 | 🗸 | 🗸 | 🗸 | 🗸 | 🗸 | 🗸 | 🗸 | 8 |
| Abou-Raya et al. 2014 | 🗸 | 🗸 | 🗸 | 🗸 | 🗸 | 🗸 | 🗸 | 🗸 | 8 |
| Petrella et al. 2002 | 🗸 | 🗸 | 🗸 | 🗴 | 🗸 | 🗸 | 🗸 | 🗸 | 7 |
| Cubucu et al. 2005 | 🗸 | 🗴 | 🗴 | 🗴 | 🗸 | 🗸 | 🗸 | 🗸 | 5 |
| Diracoglu et al. 2009 | 🗸 | 🗸 | 🗸 | 🗸 | 🗴 | 🗸 | 🗸 | 🗸 | 7 |
| Munteanu et al. 2011 | 🗸 | 🗸 | 🗸 | 🗸 | 🗸 | 🗸 | 🗸 | 🗸 | 8 |
| Saccomanno et al. 2016 | 🗸 | 🗸 | 🗸 | 🗸 | 🗸 | 🗸 | 🗸 | 🗸 | 8 |
| DeCaria JE et al. 2012 | 🗸 | 🗸 | 🗸 | 🗸 | 🗸 | 🗸 | 🗸 | 🗸 | 8 |
| Gabay C et al. 2011 | 🗸 | 🗸 | 🗸 | 🗸 | 🗸 | 🗸 | 🗸 | 🗸 | 8 |
| Brühlmann P et al. 2003 | 🗸 | 🗸 | 🗸 | 🗴 | 🗸 | 🗸 | 🗸 | 🗸 | 7 |
| Mendes et al.  2019 | 🗸 | 🗸 | 🗸 | 🗸 | 🗸 | 🗸 | 🗴 | 🗸 | 7 |
| Petterson et al.  2018 | 🗸 | 🗸 | 🗸 | 🗸 | 🗸 | 🗸 | 🗸 | 🗸 | 8 |

Was the research described as randomized? 2-Was the approach of randomization appropriate? 3-Was the research described as blinding? 4-Was the approach of blinding appropriate? 5-Was there a presentation of withdrawals and dropouts? 6-Was there a presentation of the inclusion/exclusion criteria? 7-Was the approach used to assess adverse effects described? 8-Was the approach of statistical analysis described?
