# Supplementary material for: Meta-analysis of pain and function placebo responses in pharmacological osteoarthritis trials
Source: Arthritis Res Ther. 2019 Jul 15;21:173. doi: 10.1186/s13075-019-1951-6 (PMC6631867; doi:10.1186/s13075-019-1951-6)
Supplement: Supplementary file 7 — Table S3. Summary of the outcomes reported in the included studies. (DOCX 15 kb) [file 13075_2019_1951_MOESM7_ESM.docx]

**Supplemental Table 3. Summary of the outcomes reported in the included studies.**

| Study | Outcomes | | | | |
| --- | --- | --- | --- | --- | --- |
|  | Patient self-reported Outcomes | | Subjective Outcomes | | |
|  | Pain | Function | Walk time/distance | Muscle Strength | Range of motion |
| Bucsi et al. 1998 | ✓ | ✓ | ✓ | ✗ | ✗ |
| Uebelhart et al. 2004 | ✓ | ✓ | ✓ | ✗ | ✗ |
| Fransen et al. 2015 | ✓ | ✓ | ✓ | ✗ | ✗ |
| Frestedt et al. 2008 | ✓ | ✓ | ✓ | ✗ | ✗ |
| Petersen et al. 2011 | ✓ | ✗ | ✓ | ✓ | ✗ |
| Kanzaki N et al. 2015 | ✓ | ✓ | ✓ | ✓ | ✗ |
| Messier et al. 2007 | ✓ | ✓ | ✓ | ✗ | ✗ |
| Kanzaki et al. 2012 | ✓ | ✓ | ✗ | ✗ | ✓ |
| Raynauld et al. 2003 | ✓ | ✓ | ✓ | ✗ | ✓ |
| Lambert et al. 2007 | ✓ | ✓ | ✗ | ✗ | ✓ |
| Abou-Raya et al. 2014 | ✓ | ✓ | ✓ | ✗ | ✗ |
| Petrella et al. 2002 | ✓ | ✗ | ✓ | ✗ | ✗ |
| Cubucu et al. 2005 | ✓ | ✓ | ✓ | ✗ | ✗ |
| Diracoglu et al. 2009 | ✓ | ✓ | ✗ | ✗ | ✓ |
| Munteanu et al. 2011 | ✓ | ✓ | ✗ | ✓ | ✓ |
| Saccomanno et al. 2016 | ✓ | ✓ | ✗ | ✗ | ✓ |
| DeCaria JE et al. 2012 | ✓ | ✓ | ✓ | ✗ | ✗ |
| Gabay C et al. 2011 | ✓ | ✓ | ✗ | ✓ | ✗ |
| Brühlmann P et al. 2003 | ✓ | ✓ | ✓ | ✗ | ✗ |
| Mendes et al. 2019 | ✓ | ✓ | ✓ | ✗ | ✓ |
| Petterson, et al. 2018 | ✓ | ✓ | ✗ | ✗ | ✓ |

✓=results were reported; ✗=results were not reported
