# Supplementary material for: Meta-analysis of pain and function placebo responses in pharmacological osteoarthritis trials
Source: Arthritis Res Ther. 2019 Jul 15;21:173. doi: 10.1186/s13075-019-1951-6 (PMC6631867; doi:10.1186/s13075-019-1951-6)
Supplement: Supplementary file 8 — Table S4. Summary of standardized instruments tools used for patient-reported pain and function in the included studies. (DOCX 19 kb) [file 13075_2019_1951_MOESM8_ESM.docx]

**Supplemental Table 4. Summary of standardized instruments tools used for patient reported pain and function in the included studies.**

| Study | Patients reported pain instruments | Patients reported function instruments |
| --- | --- | --- |
| Bucsi et al. 1998 | 1. VAS pain | 1. Lequesne’s Index |
| Uebelhart et al. 2004 | 1. Huskisson VAS | 1. Lequesne’s AFI |
| Fransen et al. 2015 | 1. WOMAC pain | 1. WOMAC function 2. SF-12 PCS |
| Frestedt et al. 2008 | 1. WOMAC pain | 1. WOMAC function |
| Petersen et al. 2011 | 1. VAS pain | ✗ |
| Kanzaki N et al. 2015 | 1. VAS at rest 2. VAS at walk | 1. JOA function |
| Messier et al. 2007 | 1. WOMAC pain | 1. WOMAC function |
| Kanzaki et al. 2012 | 1. JOA pain walking 2. JOA pain stairs | 1. JOA function |
| Raynauld et al. 2003 | 1. WOMAC pain 2. Physician’s global VAS assessment 3. Patient’s global VAS assessment 4. Patient’s global VAS assessment at night | 1. WOMAC function |
| Lambert et al. 2007 | 1. WOMAC pain 2. SF-36 body pain | 1. WOMAC function 2. SF-36 physical function |
| Abou-Raya et al. 2014 | 1. VAS pain 2. WOMAC pain 3. PGA VAS | 1. WOMAC function |
| Petrella et al. 2002 | 1. VAS pain 2. Self-paced walk test pain 3. Self-paced stepping pain | ✗ |
| Cubucu et al. 2005 | 1. VAS at night 2. VAS at rest 3. VAS walking 4. WOMAC pain | 1. WOMAC function |
| Diracoglu et al. 2009 | 1. VAS activity 2. VAS resting 3. WOMAC pain | 1. WOMAC function |
| Munteanu et al. 2011 | 1. FHSQ pain 2. Severity of pain during walk VAS 3. Severity of pain during rest VAS 4. SF-36 body pain | 1. FHSQ function 2. SF-36 physical function |
| Saccomanno et al. 2016 | 1. WOMAC pain | 1. WOMAC function |
| DeCaria JE et al. 2012 | 1. WOMAC pain | 1. WOMAC function |
| Gabay C et al. 2011 | 1. Patient’s assessment of global hand pain VAS | 1. Functional Index for Hand OA |
| Brühlmann P et al. 2003 | 1. VAS pain | 1. Lequesne’s Index |
| Mendes et al. 2019 | 1. VAS at rest 2. VAS at move 3. WOMAC pain | 1. WOMAC function |
| Petterson, et al. 2018 | 1. Patient global assessment VAS 2. Evaluator global assessment VAS | 1. WOMAC function |

VAS: visual analogue scale; WOMAC: Western Ontario and McMaster Universities Osteoarthritis Index; AFI: algo-functional index; SF-12 PCS: 12-Item Short Form Survey Physical Health Composite Scale; JOA: Japanese Orthopaedics Association; SF-36: 36-Item Short Form Survey; PGA: patient global assessment; FHSQ: The Foot Health Status Questionnaire; OA: Osteoarthritis
